# State of the Art and Perspectives on Silicon Photonic Switches

**DOI:** 10.3390/mi10010051

**Published:** 2019-01-13

**Authors:** Xin Tu, Chaolong Song, Tianye Huang, Zhenmin Chen, Hongyan Fu

**Affiliations:** 1 School of Mechanical Engineering and Electronic Information, China University of Geosciences, Wuhan 430074, China; tuxin@cug.edu.cn (X.T.); tianye_huang@163.com (T.H.); 2 Tsinghua-Berkeley Shenzhen Institute, Tsinghua University, Shenzhen 518055, China; zhenmin.chen@sz.tsinghua.edu.cn (Z.C.); hyfu@sz.tsinghua.edu.cn (H.F.)

**Keywords:** silicon photonics, waveguide, switch, integrated optics, MEMS, integration, actuator

## Abstract

In the last decade, silicon photonic switches are increasingly believed to be potential candidates for replacing the electrical switches in the applications of telecommunication networks, data center and high-throughput computing, due to their low power consumption (Picojoules per bit), large bandwidth (Terabits per second) and high-level integration (Square millimeters per port). This review paper focuses on the state of the art and our perspectives on silicon photonic switching technologies. It starts with a review of three types of fundamental switch engines, i.e., Mach-Zehnder interferometer, micro-ring resonator and micro-electro-mechanical-system actuated waveguide coupler. The working mechanisms are introduced and the key specifications such as insertion loss, crosstalk, switching time, footprint and power consumption are evaluated. Then it is followed by the discussion on the prototype of large-scale silicon photonic fabrics, which are based on the configuration of above-mentioned switch engines. In addition, the key technologies, such as topological architecture, passive components and optoelectronic packaging, to improve the overall performance are summarized. Finally, the critical challenges that might hamper the silicon photonic switching technologies transferring from proof-of-concept in lab to commercialization are also discussed.

## 1. Introduction

Optical switching is a very promising technology in the applications of telecommunication networks, data center networks and interconnect between multi-processes for high-performance computing [1,2,3]. In long-haul telecommunication networks, some bottlenecks of traditional electrical switches in metro-networks and backbone networks are more and more obvious with the increase of network throughput and bandwidth. Firstly, the maximum capacity of the traditional core router/switch is as high as 64 Tbps which is very challenging for ~Pbps interconnect between network nodes in the near future. Further, since the peak data rate of 5G wireless is up to 10 Gbps and the acceptable backhaul delay of 5G networks is within 1 millisecond, only direct optical connection can meet the requirement, considering the optical delay of 5 μs/km in the fiber links. In addition, the power consumption of electrical switching is up to 10 K watts which is close to the limit of thermal dissipation. So it is desirable to replace the electrical switching with all-optical switching as soon as possible to save more energy [4,5,6]. Optical switches are critical network nodes which establish point-to-point communication channels. Wavelength-selective switches (WSS) and free-space micro-electromechanical systems (MEMS) optical switching technologies have been deployed in the reconfigurable optical add-drop multiplexers (ROADMs) of the network nodes for network provisioning and patch panel interconnecting, where the typical switching time is in the order of milliseconds. 

In short-haul data center networks, the network reconfiguration is frequently required according to the dynamic workloads [7]. The high-speed electrical switches dominates the current applications, in which the input optical signals are preliminarily converted to electrical domain; then the signals go through amplification, reshaping, retiming and switching in electrical domain; finally the signals are converted back to optical domain. The optical/electrical/optical (O/E/O) conversions always require the optical converters, i.e., the transceivers, which bring additional power consumption to the system. More importantly, the electrical switching is incompatible with the wavelength division multiplexing (WDM) technology, where each optical link needs a multiplexer, a de-multiplexer and a pair of transceivers. This increases the total cost and system complexity. However, optical switching does not require O/E/O conversions and is transparent to data bit rate, signal format and protocol, with lower power consumption and lower cost. Although the speed of optical switch (i.e., from nanoseconds to milliseconds) is still slower than that of electrical switch (i.e., ~picoseconds) and all-optical signal processing is immature [8,9,10], it is possible to have both of them cooperate together to handle the switching scenarios of long packets and short burst packets based on their own advantages. 

Besides, with the advent of cloud computing, IoT (Internet of Things) and AI (Artificial Intelligence), the information switching technology for point-to-point exchange with high-speed and large-capacity data will be widely used in the high-performance data-intensive computers. The applications target at marketing, biomedicine, cyber-security, finance and national defense and so forth. [11,12]. These application scenarios require that the processors can handle a large number of random data and exploratory requests in real time, to realize nanosecond-scale switching of frequent short messages. However, the bottleneck in high-speed electrical switches has appeared due to the limited density of the pin and metal routing over the past two decades. The power consumption of the electronic chipset doubles every three years and the performance deteriorates dramatically due to the increasing temperature. To solve these problems, new microprocessor architectures based on optical switching technologies are expected to significantly improve the bandwidth and latency characteristics of on-chip interconnects. With this new technology, the total power consumption is required to be pJ-per-bit-scale for billions of floating-point operations and the cost will be in the level of ~¢/Gb/s in the future. Therefore, optical switching technology is a potential solution to address the signal configuration from long-haul networks to on-chip interconnect.

In recent years, several types of optical switches have been extensively studied, including MEMS [13], liquid crystal on silicon (LCOS) [14], LiNbO_3_ waveguide [15], III-IV semiconductor optical amplifier (SOA) [16], Mach-Zehnder interferometer (MZI) [17] and micro-ring resonator (MRR) [18]. Compared with the above technologies, the silicon photonic switches based on silicon-on-insulator (SOI) platform attract more attentions because of the following advantages: (1) high device density, whose volume is 1/1000 of silicon dioxide device; (2) functional integration with active photonic devices and complementary metal oxide semiconductor (CMOS) circuit; (3) fabrication process compatible with a mature CMOS manufacturing technology; (4) mass production with 12-inch SOI wafer, whose cost can be reduced to 1/200 of III-IV semiconductor; (5) strong thermo-optic and free-carrier plasma dispersion effects for low driving power.

Some review papers have been published on the silicon photonic switches but most of them emphasize on the electro-optic silicon switches in computer communication system [19], III-V/Si hybrid switch fabrics [20], MRR switches in datacenters [21,22] and newer switches not yet adequately explored or exploited [23]. In this paper, we systematically discuss the state of art of the silicon photonic switch engine, for example, MZI, MRR and MEMS waveguide coupler. On top of that, we perform a comprehensive review on the performance of the large-scale switch fabrics, including academic studies and industrial applications, based on the configuration of the above switch engines. We also demonstrate our latest work in the prototype of silicon photonic switches used for the telecommunication network nodes and summarize the key technologies including the topological architecture, component designs and package & control. Finally, we look into the critical challenges that need to be considered for commercialization of this technology in the future.

## 2. Silicon Photonic Waveguide Switch Engines

### 2.1. MZI Switch Engine

**Figure 1:**
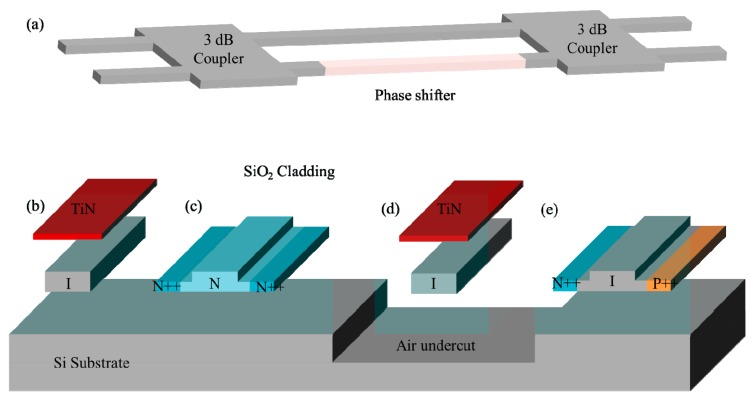
(**a**) Schematic of 2 × 2 Mach-Zehnder interferometer (MZI) switch cell. Cross-sections of waveguide phase shifters: (**b**) thermo-optic (T-O) phase shifter using a metal heater (**c**) T-O phase shifter using a doped resistive heater (**d**) suspended T-O phase shifter using a metal heater (**e**) electro-optic (E-O) phase shifter.

MZI switches are broadband interferometric switch engines, which are not limited by channel spacing and grid configuration. Hence, they are very suitable for space switching in the WDM system. A typical 2 × 2 MZI switch cell consists of two 3 dB coupler and a dual-waveguide arm between them, as shown in Figure 1a. One of the arms has a phase shifter based on the change of refractive index. Since the silicon has both strong thermo-optic (T-O) effect (1.86 × 10^−4^ K^−1^) [24] and free-carrier plasma dispersion effect [25], the phase shifter can be categorized as T-O switch with a heater and electro-optic (E-O) switch with a p-i-n junction diode. With the phase difference between the two arms changing from 0 to π, the output signal is switched from cross port to bar port due to the phase interference. The T-O switch has a response time of microsecond-scale to millisecond-scale, which fits the applications of inter-data center connect and the switching in the telecommunication network nodes. While the E-O switches have a response time of nanosecond-scale, which are good candidates for the application of intra-data center connect and interconnect between microprocessors. For the T-O switch, there are several types of heaters [26]. One employs a metal film, for example, TiN, Pt or W, placed above the waveguide, as shown in Figure 1b. When applying a current to the metal film, the Joule heat transfers to the silicon waveguide via the cladding silica and realize the modulation of temperature-dependent refractive index. The modulation efficiency is limited by the thermal conductivity of the cladding material and the spacing between the waveguide the heater. The switching power consumption is typically in the order of milliwatts and the response time is in tens of microseconds. The other one employs a rib waveguide with a doped profile N++/N/N++ in the cross-section, as shown in Figure 1c. Two heavily doped N++ regions located in the shallow-etched waveguide are used to make Ohmic contacts between the electrical wires and the silicon waveguide while the lightly doped N region is a highly resistive region for heating. This heater in physical contact with the waveguide can offer a faster and more efficient modulation due to the large overlap between the optical mode profile and the silicon heater but this configuration suffers from a higher optical absorption loss in the doping region. To further improve the T-O phase modulation efficiency, one possible method is to introduce a deep etched cavity surrounding the heater and waveguide, as shown in Figure 1d. In this way, the suspended waveguide and heater can be isolated from the cladding. The thermal dissipation path is restricted to the ground so the power consumption is reduced dramatically. On the other hand, the response time becomes slower with the increased thermal capacity due to the isolation. With this type of phase shifter, Fang et al. [27] and Chen et al. [28] achieved a switching power (response time) of 0.5 mW (0.3 ms) and 1.5 mW (0.5 ms), respectively. For the E-O switch, the phase shifter is actually a p-i-n junction diode, as shown in Figure 1e. When applying a forward bias current, the refractive index of the silicon waveguide decreases with an increase of carrier concentration and the phase shift varies as a function the applied current (carrier density). It should be noted that the modulation of carrier concentration also changes the optical absorption in the waveguide [29] and the absorption-induced power imbalance could result in both insertion loss and worse crosstalk.

**Figure 2:**
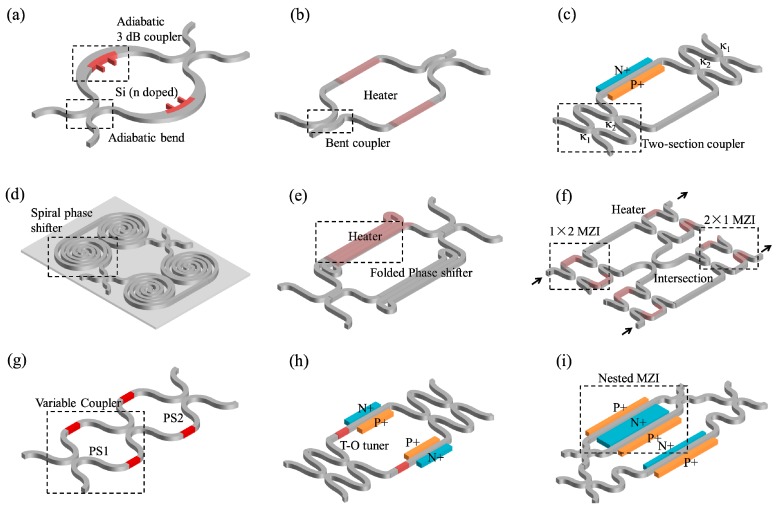
Schematic of various of MZI switch cells: (**a**) Adiabatic T-O MZI switch (**b**) T-O switch with bent couplers (**c**) E-O switch with two-section couplers (**d**) E-O switch with spiral phase shifter (**e**) T-O switch with folded phase shifter (**f**) T-O switch array (**g**) T-O switch with variable coupler (**h**) E-O switch with push-pull driving scheme (**i**) E-O switch with nested MZI phase shifter.

Figure 2 lists typical demonstrations to improve the performance of the MZI switch cell. To have a large bandwidth, several types of 3 dB couplers are employed in a MZI switch cell [30,31,32]. Watts et al. reported a compact T-O switch using an adiabatic curved phase shifter and two adiabatic 3 dB couplers (Figure 2a) [30]. With a small footprint (~10 μm) and doped silicon heater that is directly integrated to the waveguide, the switch time is as fast as 2.4 μs. Chen et al. proposed a low-loss and broadband T-O MZI switch with 2 × 23 dB couplers based on bent directional couplers (Figure 2b) [31]. The bent directional couplers have two identical uniform waveguides which makes the design and the structure simplified. Campenhout et al. presented a design of a broadband coupler, i.e., two-section (coupling ratios κ_1_ and κ_2_) rotational symmetry directional couplers, for an effective broadband 3 dB power splitting in a E-O switch cell (Figure 2c) [32]. This multi-stage coupler realized an optical bandwidth of 110 nm subject to a temperature variation up to 30 K. To obtain a low power consumption, longer phase shifters help to increase effective modulation length. Dong et al. reported an compact efficient and ultrafast E-O switch cell (Figure 2d) [33]. Owing to the 4 mm-long phase shifter in a spiral pattern, the decreased carrier density in the waveguide can reduce the free-carrier absorption and the switching power is only 0.6 mV with a low drive current of 0.72 mA. Lu et al. and Celo et al. demonstrated an ultra-efficient T-O switch cell based on the suspended folded waveguides in the phase shifter (Figure 2e) [34,35]. The new structure increases the optical interaction length of the light with the heating region and decreases the energy dissipation by using the thermal isolation. The device has an ultra-low power of 50 μW, the magnitude of an order reduction in power consumption compared to the previous reported T-O switches. To decease the optical crosstalk, four typical structures are introduced here [36,37,38,39]. Shoji et al. presented a dilated 2 × 2 MZI switch by replacing the switch with two 1 × 2 and two 2 × 1 switches so that the two optical signals can hardly interfere in the same MZI (Figure 2f) [36]. Together with a 0 dB directional coupler based intersection, the dilated switch cell has a low optical crosstalk of −50 dB and −30 dB for “bar” and “cross” switching states. This idea was also demonstrated in an E-O switch cell to obtain an optical crosstalk of −31 dB by Xing et al later [40]. Suzuki et al presented another method to achieve an ultra-low optical crosstalk of −50 dB in a 2 × 2 switch cell with a variable splitter as the front 3 dB coupler (Figure 2g) [37]. The variable coupler, which can be taken as another interferometer, has a complementary coupling ratio to the rear one, in order to minimize the overall optical crosstalk. Additionally, Dupuis et al. reported a low-crosstalk E-O switch operating at push-pull driving scheme (Figure 2h) [38]. Compared to the conventional switch cell, the push-pull driving scheme needs only π/2 per phase shifter (at π/2 bias point) rather than π in a single arm driving (at 0 bias point) to switch the constructive port to destructive port. The lower required phase difference leads to lower free carrier absorption thus a lower optical crosstalk and insertion loss. Besides, the push-pull scheme makes the insertion loss identical for both cross and bar status, which only requires a cheaper receiver with a smaller dynamic range than the single arm driving scheme. Recently, Dupuis et al. also presented a novel 2 × 2 nested E-O MZI switch with a suppressed optical crosstalk of −34.5 dB (Figure 2i) [39]. The nested MZI phase shifter in the upper arm is symmetrically driven in a push-pull scheme to vary the phase change from 0 to π with a constant insertion loss, which can be accurately balanced by a variable optical attenuator in the lower arm. Also, T-O tuners are usually integrated with the E-O phase shifter for fabrication-induced phase errors correction [38,39,41]. It should be noted that Ohmic heating generated by the applied current flow through the E-O switch also induces a phase shift due to the temperature dependent index, which counters the carrier-induced phase shift. This self-heating effect not only reduces the E-O modulation efficiency but also results in longer switching time. One idea to solve the problem is to put an E-O phase shifter within the thermal diffusion length of a T-O tuner for automatic external self-compensation, with a close loop of feedback control.

### 2.2. MRR Switch Engine

**Figure 3:**
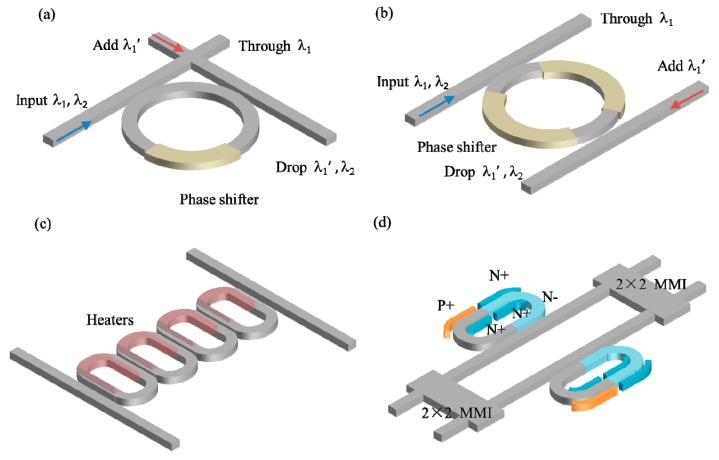
Schematic of (**a**) MRR switch with input and output crossing (**b**) MRR switch with input and output parallel (**c**) High-order MRR switch (**d**) MRR assisted MZI switch.

MRR switches are resonant switch engines, which offer a wavelength selective filtering feature for the wavelength switching applications in WDM systems [21]. Compared with the MZI switches (~mm long), MRR switches (~μm long) have more compact sizes and relatively low tuning power consumption, which are promising for building blocks of high-density switches. A typical 2 × 2 add-drop switch cell consists of a microring resonator and two bus waveguides (with a crossing in Figure 3a or parallel in Figure 3b configuration) coupled to the resonator and an optical phase shifter (either T-O or E-O actuated) in the resonator. Light that enters the input port can be switched to either through port or drop port depending on the wavelength alignment with the resonance of the MRR. When an input optical signal (λ_2_) is aligned with the resonance, it will be switched to the drop port, otherwise the light (λ_1_) will goes to the through port. New optical signal with the same wavelength as λ_1_ (denoted as λ_1_’) can be uploaded through the add port to the drop port. However, MRR switches are not widely used because of two shortcomings as follows. Firstly, the transmission spectrum of a MRR has a Lorentzian line-shape which limits the optical bandwidth and the crosstalk between adjacent channels. Secondly, the resonance wavelength of a MRR is very sensitive to the fabrication errors, laser wavelength drift and the surrounding temperature change so the wavelength alignment and stabilization are very challenging. To increase the optical bandwidth and decease the crosstalk, high-order cascaded MRRs (Figure 3c) are demonstrated by different research groups, to broaden the spectrum bandwidth and modulate the line-shape in a box-like profile [42,43,44,45,46,47]. Specifically, hitless switching, i.e., switching signal at one channel without affecting the other channels in the networks, is performed using the topology of coupled MRRs [43,47]. Recently, Lu et al. reported an E-O dual-MRR assisted MZI switch (Figure 3d) [48]. In this device, one MRR is coupled to the upper arm and operates at resonance wavelength λ_1_. The other MRR is coupled to the bottom arm of the MZI and the resonance wavelength is blue shifted to λ_2_ after tuning. Combining the merits of resonance enhancement in MRRs and the coherent interference in MZIs, slight refractive index variation of one ring induces a π phase difference between the two arms at λ_p_ = (λ_1_ + λ_2_)/2, changing the state of the switch. Therefore, this device is more power efficient than individual MZIs and MRRs. To stabilize the resonance wavelength of MRRs, two categories of compensation mechanisms are proposed. One is the passive method, in which the silicon waveguide is covered with a polymer [49,50] or TiO_2_ [51,52] with a negative T-O coefficient as the cladding to compensate the that of the silicon waveguide. The other is the active method, in which a photo-detector is used to monitor the resonance change and align the wavelength in time by tuning the E-O or T-O phase shifter as a feedback [53,54,55,56]. The active method provide a more efficient wavelength stabilization but at a cost of system complexity. In addition, new architecture based on optical mode-division multiplexing MRR switches have been proposed for on-chip communication [57,58,59]. The implementation of the multimode waveguides and mode transition between different orders allow the scaling of the bandwidth density for on-chip communication.

### 2.3. MEMS Actuated Switch Engine

**Figure 4:**
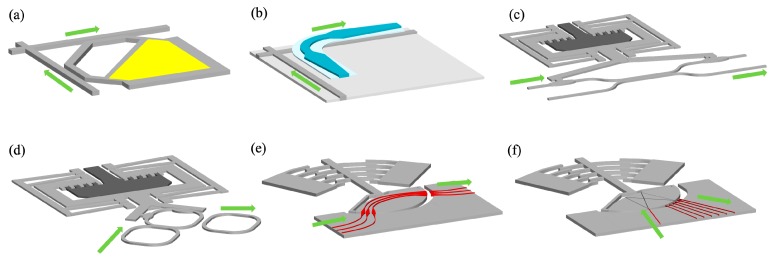
Schematic of (**a**) a switch based on vertically MEMS-actuated directional waveguide coupler (**b**) a switch based on vertically MEMS-actuated adiabatic waveguide coupler (**c**) a switch based on laterally MEMS-actuated directional waveguide coupler (**d**) a switch based on laterally MEMS-actuated MRR (**e**,**f**) a switch based on rotationally MEMS-actuated waveguide.

The MZI and MRR switches based on T-O or E-O tuning mechanisms are always prone to the fabrication errors and the complicated control of a large number of phase shifters for phase trimming. Recently, a promising mechanical tuning method, i.e., by mechanically moving or deforming waveguides, has been proposed to switch optical signals between different ports [60,61]. The new switch engines have many advantages over other methods such as a low power consumption due to the electrostatic actuation, a small footprint compatible with silicon photonic devices and digital actuation without accurate phase trimming. The response time can be reduced to the level from sub-microsecond to microsecond, which is suitable for the applications of inter-data center connect. Figure 4 illustrates configurations of various MEMS actuated silicon photonic waveguide switch engines. Han et al. reported a new type of switch based on vertically MEMS-actuated directional coupler (Figure 4a) [62]. At the initial state, the movable shunt waveguide bends upwards due to the residual stresses that are induced during gold layer deposition. The light goes to the through port directly. With an applied voltage between the shunt waveguide and the substrate, the waveguide will be pulled downwards due to the electrostatic attraction. At this state, the light is coupled to the shunt waveguide and goes out from the drop port. One year later, Seok et al. improved the switch by replacing the lateral channel waveguide directional coupler with a vertical rib waveguide adiabatic coupler in a dual-layer silicon photonic platform (Figure 4b) [63]. The improved device switches the optical signal by electrically adjusting the vertical gap between two-layer waveguides. Since the optical signal always propagates in the bottom layer waveguide unless it is required to be switched to other paths, the function of signal redirecting is decoupled from the propagation at a switch node, thus the optical switching loss and crosstalk at each stage will not be accumulated in the switch fabric. In addition, the improved switch operates at digital actuation with a stopper between the movable layer and the bottom layer, which simplifies the control and achieves a considerably low optical crosstalk. The improved switch has a faster switching time of 0.91 μs and an ultra-low crosstalk of −60 dB over 300 nm bandwidth at the applied voltage of 42 V. The through loss and drop loss are as low as 0.026 dB and 0.47 dB, respectively. Besides, MEMS-actuated switches based on directional couplers [64,65] and MRRs (Figure 4c,d) [66] are demonstrated by the researchers from Tohoku University. Unlike the MEMS-actuated switches in Figure 4a,b, these devices operate with a low-voltage comb electrostatic actuator. Recently, Briere et al. reported a MEMS-actuated switches based on the laterally rotating comb actuators (Figure 4e,f) [67]. The light turns on when the fixed port is aligned with one of the rotational ports. These devices with integrated silicon nitride waveguides on the silicon platform function as the 1 × N switches with low constant optical loss. Because of the butt-coupling scheme, the device exhibits a broadband operation. The drawback of the designs are the slow time response of (~300 μs) and high drive voltage of ~118 V due to the large mass of the movable part.

## 3. Silicon Photonic Waveguide Switch Fabrics

With the rapid development of CMOS technology and wafer fabrication process, it is coming into a reality that thousands of electronic and photonic devices can be monolithically integrated in a single chip soon. Various research groups from academic institutes and industry have demonstrated large-scale silicon switch fabrics based on the switch engines. Table 1 summarizes the typical results of the state-of-art large-scale silicon switch fabrics. Other detailed characteristics are introduced in the paragraphs below.

### 3.1. Switch Fabrics Based on MZIs

During the period from 2011 to 2015, two types of 8 × 8 T-O silicon MZI switch fabrics for the transponder aggregator (TPA) in colorless, directionless, contentionless (CDC)-ROADM applications were demonstrated by Nakamura et al. [68,69]. The switches are fabricated with 1.5-μm-thick shallow-etched rib silicon waveguides so the TE and TM modes have similar effective indices. The first demonstration of high-density silicon photonics integration is an 8 × 8 split & select T-O MZI switch fabric, which was fabricated on an 8-inch wafers with 220-nm-thick silicon using CMOS compatible processes. Variety of passive and active components, such as crossings, ridge-rib transitions, edge couplers and T-O tuners with deep etched cavities were integrated in a die [28]. Later, a monolithic 32 × 32 path-independent-loss (PILOSS) T-O MZI switch fabric with 45 nm silicon photonics CMOS process line using 12-inch SOI wafers was presented by Tanizawa et al. [70]. The switch fabric consists of 1024 switch cells and 961 waveguide crossings based on 0 dB directional couplers. Flip-chip packaged with a LGA interposer, the total footprint of the silicon photonic device is only 1/46 of a silica switch based on the planar lightwave circuit (PLC) technology. The pulse width modulation (PWM) technology is used to control the switch through a FPGA. Recently, the optical package of large number of ports are improved [71], by using a high-refractive-index PLC connector. On the other hand, the E-O MZI switch fabrics in the scalability of 4 × 4 [72] and 8 × 8 [73] in double layer topology were reported by the researchers from IBM. These switches monolithically integrate the CMOS logic, the switch driver and the photonic devices using its 90 nm silicon integrated photonics technology for the first time. Soon afterwards, a 16 × 16 Benes E-O MZI switch was illustrated by Lu et al [41]. The switch is composed of 56 switch cells, with two T-O tuners in each cell to compensate the phase errors due to the fabrication errors and temperature changes. Up to now, the largest-scale MZI switch are 32 × 32 E-O switch fabric [74] and a 64 × 64 T-O switch fabric [75], which were reported by Qiao et al. Both of them are with Benes topologies and have several power monitors inserted at the certain positions to instantaneously detect and efficiently optimize the operating points of all switch cells.

Recently we demonstrated a 32 × 32 T-O MZI switch fabric based on a new topology named hybrid dilated Benes [76]. With this unique topology, the switch fabric achieves quite low optical crosstalk but with fewer switch cells. This chip was fabricated with IME’s silicon photonic 200 nm wafer technology using 248 nm UV optical lithography, which included 448 T-O switch cells, 1856 waveguide crossings, 864 on-chip tap-monitor germanium detectors and 68 optical spot size converters. The thermal isolation technology was used to achieve a low power consumption of 1 watt for the overall chip. Also, we presented a dual-polarization 16 × 16 T-O MZI add/drop switch fabric supporting 400 Gb/s PDM-16QAM signals transmission [77]. The switches consist of the on-chip polarization splitter rotators (PSRs) [83] and dual switch cores based on the polarization diversity technology, including 416 T-O switch cells, 896 on-chip photodiode monitors, 48 PSRs and 48 spot size converters. The polarization-dependent loss (PDL), differential group delay (DGD) of express and add are 0.3 dB, <0.1 ps and 1.1 dB, <3 ps respectively. 200 Gb/s and 400 Gb/S PDM-16QAM signals were transmitted, with 0.1 dB ROSNR penalty at 200 Gb/s.

### 3.2. Switch Fabrics Based on MRRs

The first design of 5 × 5 E-O switch fabric based on MRRs were proposed by Poon et al. [44]. The radius of the MRR is 20 μm and total footprint is only 0.01 mm^2^, which is much more compact than the MZI switch fabric. The switches based on the cross-bar topology can be used for WDM system with channel spacing that agrees with the free space range (FSR) of the MRRs. Later, a broadband 8 × 7 T-O switch fabric using high-order MRRs was designed and fabricated for the first time by DasMahapatra et al. [78]. The switch employed a fifth-order-MRR switch engine with a 2-D micro-heater array which was used to locally heat up the chip to thermally tune the optical pass-band (100 GHz) of each fifth-order MRR. In 2016, a 48 × 8 T-O switch fabric based on MRRs was reported by Testa et al. [80]. This is the largest-scale MRR-based switch fabric, which aims at fabricating a highly integrated, scalable, transparent and high capacity WDM photonic switch used as a TPA in ROADM nodes. The device was designed for dropping four sets of up to 12 WDM channels and adding up to 8 WDM channels. The interleaver blocks were used to separate/recombine the input/output channels into odd and even channels and increase the wavelength spacing (200 GHz) for lower crosstalk. The switch engine is a second-order MRRs with TiO_2_ cladding which was used to stabilize the resonance wavelength. Compared with the large and expensive WSS, the new silicon photonic switch has a small volume of cubic centimeter and a cost of only hundreds of euros. Recently, a modular 8 × 8 switch fabric based on switch & select topology was proposed by Nikolova et al. [81]. This device consists of a pair of 1 × 8/8 × 1 MRR-based (de-) multiplexers with low-loss fibers or 2D optical interposer. The advantage of this design is that only two MRRs exist in each optical path and the only second-order crosstalk is allowed. However, it should be noted that the scalability is limited to high port number of the giant crossing in the central shuffle network between the (de-) multiplexers.

### 3.3. Switch Fabrics Based on MEMS Actuated Couplers

Previous silicon photonic fabric made of cascaded MZIs and MRRs have a limited port number because of the high accumulated optical loss and crosstalk. To alleviate this problem, a 64 × 64 switch fabric based on MEMS actuated couplers is demonstrated by Seok et al. [63]. This monolithically integrated silicon photonic MEMS switch used a broadband switch engine shown in Figure 4b as a building block and has quite low on-chip insertion loss from 1460 nm to 1580 nm due to the ultralow loss of multiple waveguide crossings and switching loss of only one switch cell in the cross-bar topology. The switch totally had 192 optical ports serving as input, through and drop ports which was challenging to implement the optical package in such an optical array with a large number of fibers. A 61-channel pitch reducing optical fiber arrays (PROFA) with a compact footprint of 330 μm × 280 μm were chosen to attach to the input, drop and through ports of the 64 × 64 switch fabric for high density optical packaging [84]. As for the electrical package, there are 4096 electrical interconnects (excluding grounds) and it was quite difficult to connect such a high density electrical contacts in a small area. A row-column addressing method with only 2N electrical contacts based on inherent hysteresis of the electrostatic parallel plate actuator, was demonstrated for a N × N switch fabric previously [85]. However, this drive method has a tradeoff between the contact number and switch time due to the sequential addressing. Only a sub-array of 12 × 12 switch fabric with 146 electrical contacts was flip-chip-bonding packaged with an aluminum nitride interposer as an intermediate substrate between the device the PCB board [86,87]. The final solution for 4096 electrical interconnects has not been reported. Recently a 128 × 128 silicon photonic MEMS switch was reported [82]. Compared with the 64 × 64 switch fabric, the new device has a drive voltage changed from 40 V to 25 V but higher insertion loss because of longer routing waveguide and higher propagation loss. In addition, a 50 × 50 polarization-insensitive silicon photonic MEMS switch fabric was demonstrated [88]. It had polarization-dependent components such as waveguide crossings and PSRs removed by multi-level waveguide crossbar and no dual switch cores are required to realize the polarization diversity. The measured PDL is as high as 8.5 dB (simulation result is <1 dB) and the polarization-dependent delay is 44 ps. It is believed that the total on-chip loss will be less than 2 dB with improved fabrication in the future.

## 4. Key Technologies of Silicon Photonic Switch Fabrics

In general, silicon photonic waveguide devices work well for broadband applications. The commercial deployment of silicon photonic modulator is primarily based on MZIs because it is difficult to stabilize the resonant wavelength of a MRR due to the manufacturing variability. Silicon photonic MEMS switches have excellent performance in optical loss and crosstalk but are in lack of low-voltage drive and high-density electrical package. In addition, silicon photonic (de-) multiplexers have poor wavelength tolerance for dense WDM systems so our research group targets a large scale broadband silicon photonic switch fabric based on MZIs which is expected to be a potential technology in the commercial deployment in a near future. In this section, we particularly review the key technologies in large-scale switch fabrics.

### 4.1. Switch Network Topologies

**Figure 5:**
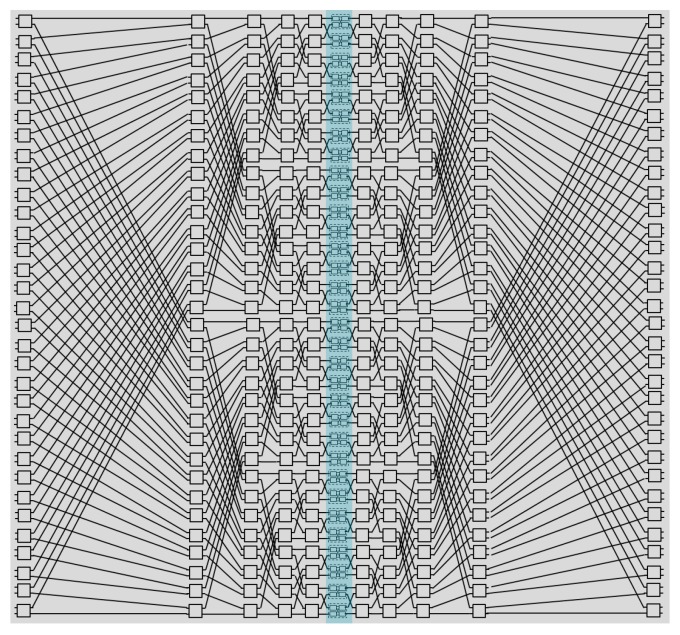
Topology of 32 × 32 Hybrid Dilated Benes.

**Figure 6:**
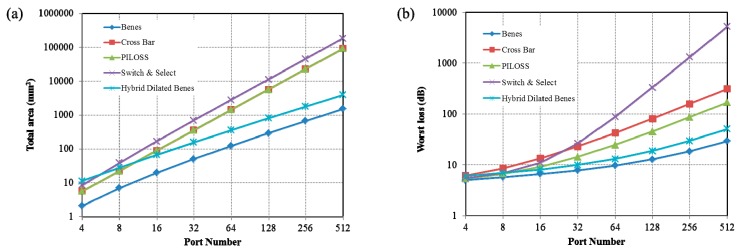
Switch matrix of different topologies (**a**) total area (**b**) insertion loss.

The network topologies play a critical role in the overall performance of the switch fabric. For an N × N optical switch fabric, the good topologies should have the features as follows: (1) a small number of switch cells and compact footprint; (2) low insertion loss based on a few switch stages; (3) high signal-to-noise ratio (SNR) due to suppressed crosstalk. There are four most widely used topologies as Benes [74], cross-bar [63], PILOSS [70] and switch & select [28], in which the first is a reconfigurable, non-blocking architecture while others are strictly non-blocking ones. Benes fabric requires the minimum number of 2 × 2 switch cells (i.e., smallest chip area) and PILOSS fabric features the advantage of almost the same optical loss in each path. However both of them have an unacceptably large first-order crosstalk for the connection states where a 2 × 2 switch cell is shared by two signals. The cross-bar fabric has a very simple configuration but the optical loss varies dramatically in different optical paths, resulting in undesirable large output power fluctuation. The switch & select fabric has the first-order crosstalk suppressed. So, its crosstalk is limited by the second-order crosstalk and that of a waveguide crossing. But the drawback is weak scalability due to the large number of switch cells. Recently, a new switch topology named hybrid dilated Benes achieves highly reduced output crosstalk with a relatively small cell number [89]. A 32 × 32 hybrid dilated Benes fabric is shown in Figure 5, consisting of an ingress column of 1 × 2 cells and an egress column of 2 × 1 cells. Compared with the previous dilated Benes topology [90], this new topology has the central column of 2 × 2 cells each replaced by a 2 × 2 switch & select fabric. In addition, the number of 2 × 2 cells for sharing two signals can be optimized using our intelligent wavelength constraint routing algorithm. And the crosstalk can be suppressed with an acceptable blocking rate of < 10-6. Figure 6 presents the comparison of chip area and insertion loss of various topologies as a function of the port number. In the estimation of chip size, we used a typically average footprint of ~0.07 mm2 for a single switch cell and assumed that the area of the switch cells, waveguide routing and optical/electrical package contacts accounted for 20%, 40% and 40% of the overall area, respectively. In the calculation of optical loss penalty, we used a typical insertion loss of 0.3 dB and 0.02 dB for a typical MZI switch cell and waveguide crossing, respectively. Coupling loss was assumed to be 2 dB per facet and propagation loss of the routing waveguides was neglected. These values can represent the performance of state-of-the-art silicon photonic devices using optical lithography. The results reveal that the hybrid dilated Benes fabric has distinct advantage compared with other topologies when the port number is larger than 32. With a reticle size of 24 mm × 32 mm in the current mainstream foundry, a 128 × 128 hybrid dilated Benes fabric with about 2300 switch cells can be fabricated.

### 4.2. Passive Components for Low Insertion Loss

**Figure 7:**
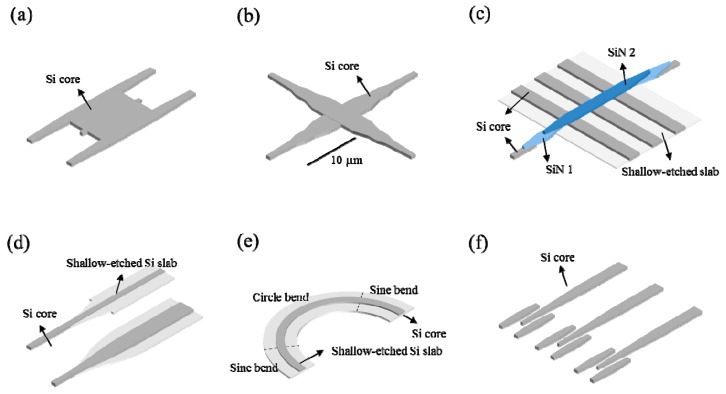
Passive components (**a**) MMI coupler (**b**) In-plane waveguide crossing (**c**) 3D waveguide crossing (**d**) transition waveguides (**e**) adiabatic bend waveguide (**f**) trident edge coupler array.

The insertion loss and bandwidth of a switch fabric are always limited by the performance of passive components. Although some research groups compensated the on-chip loss of the optical links by integrating semiconductor optical amplifiers [19,91,92,93], the deterioration of SNR due to the spontaneous emission noises is unavoidable. Therefore, scaling to a larger switch size requires an improvement of the individual passive component not only in the fabrication process but also the designs. The overall loss penalty of a switch fabric is largely dependent on the insertion loss from the switch cells, waveguide crossings, fiber coupling loss and various routing waveguides such as bends and taper transitions. 3 dB coupler is the key component in a T-O switch because its excess loss dominates the switch loss and the imbalance of the two output ports affects the control accuracy. A 1 × 2 MMI coupler [94] and adiabatic coupler [28] was reported to has an excess loss of 0.1 dB and 0.15 dB over C band. Recently we demonstrated an excellent T-O switch cell with a low loss of < 0.5 dB and crosstalk of <−30 dB, in which the MMI 2 × 2 coupler (Figure 7a) loss is <0.15 dB and imbalance is <0.3 dB cross 200 mm wafer using 248 lithography. Waveguide crossings are critical components which are usually employed in a large number in an optical path. An insertion loss of 0.02 dB [95] and 0.04 dB [28] were achieved with a fully-etched and shallow-etched waveguide, respectively. We presented a waveguide crossing (Figure 7b) with the ultralow loss of 0.007 dB and an ultralow crosstalk of −40 dB based on the three-mode synthesis of a 1-D Gaussian beam [96]. Recently, a monolithically integrated multilayer silicon nitride-on-silicon waveguide crossing (Figure 7c) was proposed [97,98] with insertion loss as low as 0.003 dB and crosstalk of −56 dB using the low-pressure-chemical-vapor-deposition (LPCVD) silicon nitride layer on SOI wafers. Routing waveguide propagation is also important. A ultra-low propagation loss of 0.5 dB/cm for a single-mode waveguide was achieved by Tanizawa et al. with 45 nm immersion lithography and 300 nm SOI wafer technologies [70]. In our chips, the propagation loss of a 500-nm-wide single mode strip waveguide is achieved to be 2.5 dB/cm, by using 193 nm and 248 nm lithography in the silicon photonic foundry of Institute of Microelectronics, Singapore. To reduce the on-chip insertion loss, 3 μm-wide multimode rib waveguides with a lower propagation loss of 0.5 dB/cm was used to connect switch cells. A 25 μm-long taper shaped as a stretched sinusoid with 0.03 dB transition loss and a 10 μm-long adapter of concave elliptic shape with 0.002 dB transition loss were proposed to connect the single-mode strip to multi-mode rib waveguide and single-mode rib waveguide, respectively (Figure 7d) [99]. We also achieved a 0.008 dB-loss rib waveguide bend by optimizing the profile in a sine-circle-sine design (Figure 7e) [100]. Trident edge couplers were employed for light input and output coupling because they have low PDL and large tolerance to the fiber misalignment (Figure 7f) [101].

### 4.3. Packages and Controls

Unlike the optical transceivers, the optical switch chips require large-scale, high-density control, optical and electrical package. We demonstrated a prototype of fully-assembly, fully-functional T-O 32 × 32 hybrid dilated Benes silicon photonic switch fabric [76,102,103]. For the optical package of high-port fiber array, there are several potential methods including the mechanically compliant evanescent polymer coupler [104,105,106], the hexagonal PROFA [84], the extremely-high-Δ (5.5%) PLC fiber array connector [71] and the silica mode field adapter/fiber spacing concentrator (FSC) [103]. We used this PLC FSC connector to fan out the pitch of 20 μm to the fiber pitch of 127 μm of a 68-core polarization maintaining fiber array with a low loss of 3.2 dB. For the electrical package, a conventional cavity-up active-side-up electrical package design was used to handle 1560 electrical I/O ports in a custom ceramic ball grid array (BGA) carrier by wire-bonding technology. The thermal management of the chip is implemented by attaching CuW substrate and thermo electric cooler (TEC). With the number of electrical contacts increasing, the flip-chip [70] and through-silicon vias (TSV) technology [107] are more suitable because they can separate the electronics from the photonics and eliminate the effects of the electrical package on the optical signal. Calibration using extra optical ports need expensive test equipment, disrupts manufacturing flow and does not allow in-service verification. We used germanium detectors in each switch cell for transient adjustment in addition to the calibration of the initial states. Commercial CMOS A/D chips were used to read all photocurrents to a controller FPGA and control the switch states with optimized crosstalk.

## 5. Discussions and Outlook

Despite of the rapid development of silicon photonic switches, further research work is required to solve following technical challenges. Firstly, most of the current silicon photonic switches use 220 nm-thick silicon waveguide, which is a highly polarization-sensitive platform. Therefore, the silicon photonic switches usually work for single polarization, which limits the switch capacity. To solve the problems, the polarization diversity scheme [77,108], polarization-insensitive micro-scale rib waveguide [68,109], on-chip polarization controller [110,111] and bidirectional port assignment in a PILOSS switch fabric [112] have been proposed to implement the polarization management. However, these methods result in neglected PDL and DGD that cannot be neglected for a large-scale switch fabric. Secondly, the photonic packet switching for interconnect potentially requires that the energy per bit is lower than 1 pJ. For our 32 × 32 hybrid dilated Benes silicon photonic switch fabric, the energy per bit is about 0.8 pJ for a 100 Gb/s light path when the switch cell power is <5 mW (1 mW for switch drive, 2 mW for T-O compensation and 2 mW for thermal management), which is dominated by T-O phase compensation and overall thermal management. However, the energy per bit will exceed 1 pJ when SOAs are integrated on chip to compensate the optical insertion loss. It is expected to further decrease the energy per bit with improved chip uniformity and low-loss passive devices. Thirdly, most switches are wire-bonded or flip-chip packaged in a ceramic carrier and controlled by the A/D chips and FPGA board. This system assembly is usually bulky and the switch port is limited by the electrical contact density. Thanks to the compatibility to CMOS process, research on AISC logic drivers monolithically integrated with photonics has been started, which will potentially benefit cost-effective intelligent high-speed control [113]. Limited by the current development of the nanofabrication technologies, the state-of-art silicon waveguide photonic switches have around 64 × 64 [75] to 128 × 128 [82] ports, each having a throughput of 400 Gb/s or higher [114]. Thus, the overall bandwidth of a monolithic switch chip can reach tens of Terabits per second. In general, silicon photonic switches will have a great progress in the future and they will be promising technologies in the applications of telecommunication networks, high performance datacenters and data-intensive computing.

## 6. Conclusions

In this paper, we have reviewed the state of the art and given our perspectives on the silicon photonic switch technologies. The optical switch has potential advantages in broadening transmission bandwidth, lowering cost and saving power energy. We gave an overview of the silicon photonic fabrics based on MZIs, MRRs and MEMS-actuated couplers. The key technologies including topologies, component designs and package & control are discussed. Finally, the technical challenges and outlook were investigated and discussed. 

## Figures and Tables

**Table 1:** Comparison table of large-scale optical waveguide switch fabrics.

Ref.	Institution	Switch Engines	Scalability & Topology	On-chip Insertion Loss (dB)	Coupling Loss (dB)	Crosstalk (dB)	Switch Time	Power (W)	Size (mm^2^)
[68]	NEC	T-O MZI	8 × 8 PILOSS	-	-	−25	-	-	12 × 3
[69]			8 × 8 Switch & select	4	1	−35	150 μs		12 × 14
[28]	Bell Labs		8 × 8 Switch & select	4	3.5	−30	250 μs	0.07	8 × 8
[70,71]	AIST		32 × 32 PILOSS	8.4	1.4	−35	30 μs	1.9	25 × 11
[72]	IBM	E-O MZI	4 × 4	3.7	-	−15	5 ns	0.05	0.165
[73]			8 × 8 Double Layer	-		-	-	-	0.675
[41]	SJTU		16 × 16 Benes	14	5	−10	3.2 ns	1.2	10.7 × 4.4
[74]	CAS	T-O MZI	32 × 32	18.5	5	−15	1.2 ns	0.54	12.1 × 5.2
[75]		E-O MZI	64 × 64 Benes	12		−30	-	-	21.7 × 9.6
[76]	Huawei	T-O MZI	32 × 32	13	3.2	−20	1.4 ms 70 μs	1 20	12 × 12
[77]			16 × 16 Hybrid Dilated Benes	22	4.5	-	-	-	12.5 × 12.5
[44]	HKUST	E-O MRR	5 × 5 Cross-bar	-	-	−11	1.3 ns	-	0.1 × 0.1
[78]	TU/e	T-O MRR	8 × 7 Cross-bar	22	6	−20	17 μs	-	-
[79]	CAS		4 × 4	-	-	−13	25 μs	-	-
[80]	Ericsson		48 × 8 Cross-bar	~3	3.2	−23	4 μs	-	8.4 × 7.8
[81]	Columbia University	T-O MRR	8 × 8 Switch & select	10	-	−39	20 μs	-	-
[63]	UC	MEMS	64 × 64	3.7	6.5	−60	<1 μs	40V	8.6 × 8.6
[82]	Berkeley		128 × 128 Cross-bar	22.7	-			25V	16 × 17
